# Evolving Concepts: How Diet and the Intestinal Microbiome Act as Modulators of Breast Malignancy

**DOI:** 10.1155/2013/693920

**Published:** 2013-09-25

**Authors:** Iuliana Shapira, Keith Sultan, Annette Lee, Emanuela Taioli

**Affiliations:** ^1^Monter Cancer Center, Don Monti Division of Oncology and Division of Hematology, Hofstra North Shore Long Island Jewish School of Medicine, 450 Lakeville Road, Lake Success, NY 11042, USA; ^2^Hofstra North Shore Long Island Jewish School of Medicine, Division of Gastroenterology, Hepatology and Nutrition, North Shore University Hospital, 300 Community Drive, Manhasset, NY 11030, USA; ^3^Feinstein Institute for Medical Research, Robert S. Boas Center for Genomics and Human Genetics and Elmezzi Graduate School of Molecular Medicine, Hofstra North Shore Long Island Jewish School of Medicine, 350 Community Drive, Manhasset, NY 11030, USA; ^4^Population Health-Hofstra North Shore-LIJ School of Medicine and North Shore/LIJ Health System, 175 Community Drive, Room 203, Great Neck, NY 11021, USA

## Abstract

The intestinal microbiome plays an important role in human physiology. Next-generation sequencing technologies, knockout and gnotobiotic mouse models, fecal transplant data and epidemiologic studies have accelerated our understanding of microbiome abnormalities seen in immune diseases and malignancies. Dysbiosis is the disturbed microbiome ecology secondary to external pressures such as host diseases, medications, diet and genetic conditions often leading to abnormalities of the host immune system. Specifically dysbiosis has been shown to lower circulating lymphocytes, and increase neutrophil to lymphocyte ratio, a finding which has been associated with a decreased survival in women with breast cancers. Dysbiosis also plays a role in the recycling of estrogens via the entero-hepatic circulation, increasing estrogenic potency in the host, which is another leading cause of breast malignancy. Non-modifiable factors such as age and genetic mutations disrupt the microbiome, but modifiable factors such as diet may also lead to profound disruptions as well. A better understanding of dietary factors and how they disrupt the microbiome may lead to beneficial nutritional interventions for breast cancer patients.

## 1. Introduction

The human digestive tract is known to host trillions of microbes collectively called the intestinal microbiota [1–4]. A commensally, mutually beneficial relationship exists between the human host and these microbiota. The host's digestive tract provides the nutrient niche for the microbiota, while the microbiota protects against pathogens, helps in the development of the immune system, aids in nutrient reclamation from food by fermenting indigestible fiber to short chain fatty acids, produces essential amino acids and vitamins, helps in the absorption of minerals, and aids the breakdown of dietary toxins and carcinogens [1–3, 5]. The intestinal microbiota also helps the growth and differentiation of enterocytes and colonocytes, thus maintaining the intestinal barrier against potential pathogens [6]. 

## 2. Origin of Microbiome

The intestinal microbiota is maternally inherited at birth as the newborn is delivered through the vaginal canal [7–9]. Later in development, factors both dependent on host choices such as diet and independent of host choices such as genetics and age modify the intestinal microbiota [10]. 

**Figure 1:**
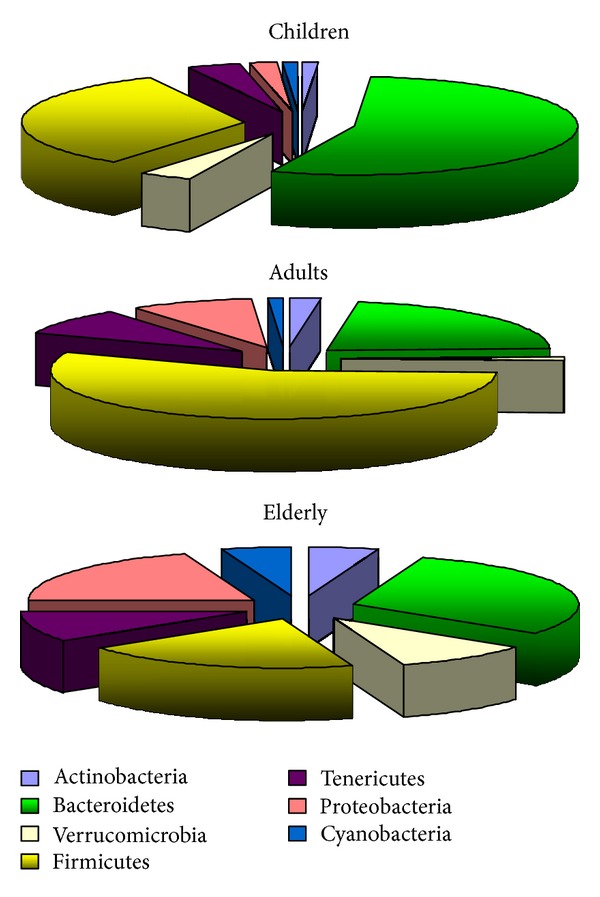
Major phylums in children, adults, and elderly detected by pyrosequencing of 16S ribosomal RNA genes [15, 16].

Insights into the dynamic structure of intestinal microbiota have become possible with the advent of next-generation sequencing technologies; such technologies are able to fully characterize the polymorphism of bacterial communities inhabiting the human intestines using bacterial 16S RNA ribosomal sequences [11–13]. This approach showed that the major phylums in adults are Firmicutes and Bacteroidetes representing over 80% of the colorectal intestinal microbiota. Minor phylums such as Verrucomicrobia, Actinobacteria, Proteobacteria, Tenericutes, and Cyanobacteria represent the remaining 20% of the colon microbiota [14] (Figure 1). 

The ratio of Firmicutes to Bacteroidetes changes with age. Newborns and infants have more Bacteroidetes; in adults Firmicutes are the predominant intestinal microbiome phylum, and in the elderly more *Bacteroides* and increased proportion of the minor phylum Proteobacteria are observed [10]. Major species associates with respective phylums are described in Table 1.

## 3. Host Influence on the Microbiome

Environmental and external factors may act on the composition of the microbiome. The term dysbiosis defines the disturbed microbiome ecology which may be secondary to external factors such as disease, medications, and diet as well as to nonmodifiable genetic conditions [18]. Knocking down genes of the immune system may lead to profound modifications in the intestinal microbiome and to predisposition to diseases of the immune system as well as cancer [19–21]. Animal models of fecal transplant of “dysbiotic” microbiome have been shown to alter the structure of recipient microbiome and to induce the development of the same immune disease that was initially seen in the genetically altered animal [19–22]. 

In general, several studies have shown that the microbiome structure suffers if genetic defects interfere with the normal development of the immune system of the animal [23–31].  Table 2 provides a summary of the current knowledge of how defects in the genetic factors that regulate the development of the immune system stunt the structure of the intestinal microbiome. 

Human research has shown that patients with mutations in Crohn's disease genes such as nucleotide binding oligomerization domain 2 (NOD2) [33] and autophagy related protein 16L-1 (ATG16L1) [34] have decreased numbers of the Firmicutes species of bacteria *Faecalibacterium prausnitzii* and *Roseburia intestinalis* in their ileum [35]. This decrease in commensal bacteria is associated with a proportional increase in the pathogenic adherent invasive *Escherichia coli* and *Salmonella typhimurium* [36, 37]. The inability of *Faecalibacterium* and *Roseburia* to survive in the intestinal microbiome of patients with Crohn's disease deprives these patients of bacteria which produce short chain fatty acids (SCFA) used as nutrition and energy for colonocytes [38]. Decreased SCFA leads to increased colonocyte death, increased cell turnover and may explain in part the 3.2-fold higher lifetime risk of colorectal cancer observed in Crohn's when compared to general population [39, 40]. 

## 4. Diet, Microbiome, Phytoestrogens, and Breast Cancer

Fiber represents the indigestible portions of plant cell walls and may be either soluble or insoluble. The insoluble fibers, such as lignins, cellulose, dextrins, waxes, and chitins are not fermented by the human commensal microbiota. Soluble fiber such as inulin, arabinoxylans, pectins, beta-glucans, amylase resistant starches, fructans, and lignans are fermented by the intestinal microbiota into short chain fatty acids [41]. Lignans are substances found in whole grains, soy, fruits, and vegetables, while inulin, arabinoxylans and oligofructose are fibers found in artichoke, onion, and banana. Pectins and fructans are found predominantly in fruits [42]. Beta-glucans are soluble fibers found in mushrooms and amylase-resistant starches are found in beans and chickpeas [43–53]. 

The presence of high concentrations of soluble fiber in the distal ileum and colon favors the growth and maintenance of beneficial *Bifidobacterium* (from Actinobacteria phylum) the anti-inflammatory *Faecalibacterium prausnitzii* (a Firmicute) as well as commensal species from Bacteroidetes phylum [54, 55]. By-products of bacterial fermentation of soluble fibers are short chain fatty acids (acetate, propionate, and butyrate), the preferred source of energy for colonocytes [56]. 

In one study, fourteen healthy volunteers consumed for 2 weeks a high fiber diet (19 grams/day) followed by 2 weeks of a low fiber diet (under 5 grams/day). The microbiome was analyzed and *Faecalibacterium prausnitzii*, *Roseburia intestinalis* (from Firmicute phylum) were quantified by 16S ribosomal tagged probes by fluorescent in situ hybridization concomitantly with the quantity of short chain fatty acids (SCFA) per gram of dry fecal weight [57]. The low fiber diet was associated with a statistically significant 80% reduction in *Faecalibacterium prausnitzii*, *Roseburia intestinalis,* and SCFA (*P* < 0.001). At 2 weeks, the fiber rich diet increased ten-folds the quantity of SCFA and of both *F. prausnitzii* and *Roseburia* (*P* < 0.01) [57]. 

The promotion of growth of Firmicutes and Bacteroidetes within the intestinal microbiome by dietary fibers has another important effect: these bacteria are able to metabolize dietary lignans into the potent phytoestrogens enterodiol (END) and its oxidation product enterolactone (ENL), which are then readily absorbed into bloodstream [58, 59]. 

Antibiotic use influences the bioavailability of enterolactone due to disruptions in the intestinal microbiome [60]. 

Unlike estrogen, high blood levels of phytoestrogens have been shown to be inversely associated with risk of breast cancer in epidemiologic studies [61, 62]. In a population based case-control study of over 6,000 women, consumption of lignans three times per week was associated with a 50% reduction in breast cancer risk in premenopausal women. Notably, this benefit was also seen in overweight and obese women [63].

Research on more than 35,000 women participants in the UK Women's Cohort Study showed that premenopausal women who ate most of their fiber from whole grains (at least 13 grams per day of whole grain fiber) and 30 grams per day of total fiber had 50% less breast cancer. If the majority of soluble fiber came from fruits rather than whole grains the protection from breast cancer was slightly lower 35% for those women who ate 30 grams of fiber compared to those who ate only 2 grams of fiber daily [64]. 

The benefit of high fiber diets extends to postmenopausal women, despite the fact that with age the intestinal microbiota ratio of Firmicutes to Bacteroidetes phylums changes in an unfavourable direction [10]. 

A study of over 51,000 postmenopausal women followed over more than 8 years showed that women who consumed 30 grams or more of fiber from fruit and whole grains had 34% less breast cancer than those who consumed less quantities of those items. Notably, lignan fiber seemed to be more protective than fruit and vegetable fibers [65]. 

In another study, premenopausal women at risk for breast cancer because of benign breast disease (such as ductal hyperplasia, lobular hyperplasia, radial scar) were asked to undergo a baseline periareolar fine needle aspiration (RPFNA) and were then followed on a diet which included daily plant lignans (intervention) for one year. Serum enterolactone was measured before and after the intervention. After intervention serum enterolactone increased ninefold from the baseline. After one year, a repeated RPFNA showed atypia in only half of the women treated with diets rich in lignans in whom the serum enterolactones increased ninefold from baseline levels [66]. 

A Finnish case-control study also looked at serum levels of enterolactone and their association with breast cancer. Women with the highest dietary intake on lignans (at least two slices of rye bread per day) were in the highest quintile of serum enterolactone (54 nmol/L) while with the lowest consumption were in the lowest quintile (3 nmol/L). After eight years of followup, women in the highest quintile of enterolactone had 62% less breast cancer diagnosed compared to the lowest quintile [67]. 

In a study of more than 300 women diagnosed with breast cancer, serum enterolactone was used to quantify the amount of dietary lignans consumed. At a median followup of 276 months there was 30% decrease in breast cancer specific mortality in patients with enterolactone levels more than 10 nmol/L [68]. 

A recent meta-analysis of serum enterolactones and breast cancer showed a protective effect for breast cancer for women with serum levels in the highest quartile compared to the lowest (meta-OR: 0.72; 95% CI: 0.55–0.88) [69]. The effect was more pronounced in postmenopausal women [69]. 

Serum enterolactone, a product of microbiome fermentation of dietary lignans, appear to have protective effects in breast cancer patients and in breast cancer prevention. 

## 5. Diet, Microbiome, Estrogens, and Breast Cancer

Saturated fats are the source of cholesterol used by the ovaries for estrogen synthesis. In postmenopausal women the adipose tissue, adrenal glands, and other organs transform circulating androgens into estrogens using the aromatase enzymes [70]. In the blood, estrogens circulate either bound to proteins or free. The liver inactivates estrogens by conjugation, that is, sulfonation, methylation, and glucuronidation reactions; conjugated estrogens are then finally excreted with bile acids and are transported into the intestinal lumen [71]. Once in the intestinal lumen, the fate of these conjugated estrogens depends on the composition of the intestinal microbiota present in the host. Individuals with an intestinal microbiome capable of deconjugating estrogens will reabsorb the free estrogen via the enterohepatic circulation, increasing the estrogenic potency in the host. Those with an intestinal microbiome less favorable to deconjugation will promote estrogen excretion in feces [72]. Thus diet plays an important role in creating the microbiome environment that deconjugates estrogens or is indifferent to conjugated estrogens.

Diets rich in fats and red meat promote the production and excretion of bile acids necessary for fat digestion and absorption. Commensal bacteria then break down these bile acids into deoxycholic and lithocholic acids, metabolites that favor the growth of Proteobacteria species such as *E. coli*, *Klebsiella*, *Enterobacter*, and *Citrobacter* and are detrimental to certain species of Firmicutes and Bacteroidetes in the intestinal microbiota. This process in turn produces a dysbiotic state [73] that favors the growth of *E. coli* from Proteobacteria phylum, an organism that is able to produce potent beta-glucuronidases [74], deconjugating estrogens in the intestinal lumen and thus contributing to the higher estrogenic burden of the host [75]. A direct relationship between higher circulating estrogen levels and the increased risk of postmenopausal women developing breast cancer has been extensively reported in the literature [76]. 

## 6. The Microbiome's Role in Obesity and Breast Cancer 

Diet-induced obesity changes the balance of Firmicutes to Bacteroidetes phylums. Studies in twins, of which one is obese, the other nonobese, have shown smaller Bacteroidetes phylums and more Firmicutes in the obese twin as compared to the lean twin [77, 78]. Notably, the ratio of Bacteroidetes increases at the expense of Firmicutes when the obese individual loses weight or after gastric bypass surgery [79]. It is unknown whether the change in proportion of Firmicutes to Bacteroidetes, allowing for the growth of detrimental species in the microbiome, is merely an effect of obesity or a cofactor that promotes obesity. It has been speculated that intestinal dysbiosis (perpetuated by atherogenic, western diet) and not merely high caloric intake is a main cofactor in the obesity epidemic in the Western world [80]. This concomitant presence of dysbiosis and obesity, and the resulting increased circulating estrogen levels, may synergistically contribute to the 20% higher risk of breast cancer seen among women with a BMI greater than 30 kg/m^2^, as compared to normal weight controls [81]. 

## 7. The Microbiome's Role in Immune Modulation and Breast Cancer

Much of what we know about the symbiotic interaction between the intestinal microbiome and systemic immune system development has come from the study of germ-free (knows as gnotobiotic) rodents. Lack of intestinal microbiota in gnotobiotic rodents decreases the size of the small and large intestine Peyer's patches of the spleen and also affects distant immune organs. The size of the pancreas and the number of beta-cells are decreased [82], and the hypothalamic-pituitary-adrenal axis stress response is also altered [83]. The lack of a microbiome decreases the number and function of neutrophils due to decreased microbiota-derived peptidoglycans responsible for serum and bone marrow neutrophil function [84]. 

The colonization of gnotobiotic rodents with known strains of human microbiome allows measuring the effects of known bacterial species on the immune system development. 

Normal microbiome contributes to maturation of effector CD8+ T cells (known as killer T cells) via contact with *Sphingomonas* species (of Proteobacteria phylum) [85]. Inflammation decreases the proportion of *Sphingomonas* in the Proteobacteria phylum and prevents proper development of CD8+ antitumor cytotoxic T cells [86, 87]. CD8+ T cells are the most potent immune cells capable of eliminating foreign antigens and breast tumor cells [88]. 

**Figure 2:**
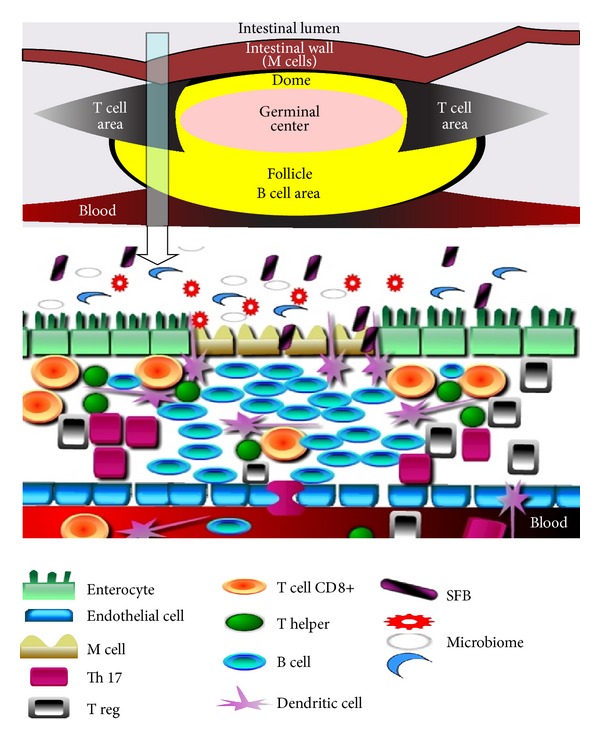
Schematic representation of Peyer's patch organization (also known as gut-associated lymphoid tissue—GALT) shows that the bulk of the tissue is made up by B cells organized in a large and highly active domed follicle. T cells occupy the areas between the follicles. The antigen enters across a specialized epithelium made up of so-called multifenestrated (M) cells. The germinal center is located in the center of the follicle. Cross-section through the Peyer's patch shows the types of cells and the interactions between the cells of the immune system and the microbiome. M cells: multifenestrated cells; Th-17: T cell helper 17; Treg: T regulatory cells; T cells CD8+: effectors T cells; T helper: naïve CD4+ T cells; B cell; SFB: segmented filamentous bacteria.

T cell differentiation takes place in the thymus from 12 weeks of gestation until the thymus regresses through involution by 9 months of age [89]. The role of thymus in immune reconstruction is replaced in part by the interactions between the trillions of organisms in the microbiome and cells of the immune system [89, 90]. These interactions take place with the help of multifenestrated epithelial cells (M cells) lining the Peyer's patches (Figure 2). Dendritic cells in the Peyer's patches sample via direct contact the microbial contents of the intestines and adapt the immune responses to the antigenic load. Segmented filamentous bacteria (SFB) have direct contact with dendritic cells in the Peyer's patches. They are necessary and sufficient to contribute to the maturation of CD8+ effector cytotoxic T cells and CD4+ helper cells [91] (Figure 2).  Table 3 summarizes the contribution of different phylums and species in the microbiome to the maturation of the immune system. 

It was previously noted that diets rich in fats and red meats result in dysbiosis, favoring the growth of Proteobacteria phylum (species such as *E. coli*, *Klebsiella*, *Enterobacter*, and *Citrobacter*) and *Fusobacterium nucleatum* [103, 104] and impacting in a negative way certain species of Firmicutes and Bacteroidetes [73]. Notably, *Fusobacterium nucleatum* species are able to kill maturing lymphocytes via M cells in the Peyer's patches via direct contact, lowering the number of circulating systemic lymphocytes [92, 105] (Figure 2). 

An intestinal microbiome that destroys lymphocytes may influence the outcomes of cancer. Patients with a lower number of systemic lymphocytes at diagnosis appear to have poorer cancer-related outcomes. Studies show that the ratio of neutrophils to lymphocytes (calculated as neutrophil count divided by lymphocyte count) at diagnosis predicts long-term cancer outcome, with higher ratios predicting worse outcomes independently of patients' age or stage at diagnosis [106–109]. 

In a study of early stage breast cancer (stages I, II, and III) a neutrophil-to-lymphocyte ratio of more than 2.5 was associated with 4-fold risk of disease relapse at 10 years compared to patients with a ratio lower than 2.5 regardless of stage or age at diagnosis (*P* < 0.001) [110]. A retrospective study of 316 breast cancer patients showed that a neutrophil-to-lymphocyte ratio of more than 3.3 at diagnosis had a 44% higher risk of death within 5 years of cancer diagnosis compared to those with a ratio less than 1.8 (*P* < 0.0001) [111]. 

In BIG-02-98 study of more than 2000 patients with node positive breast cancer more than 50% infiltration of tumor stroma with lymphocytes was associated with reduced risk of breast cancer relapse and death. For each additional 10% increase in percentage of lymphocytes the risk of relapse was 17% lower and risk of death was 27% lower independent of stage at diagnosis and patients' age (*P* < 0.0001) [112]. 

Most chemotherapy decreases tumor burden by 1-2 logs. The decrease is followed by immune recognition of tumor antigens, with the desirable response being that the rest of the tumor burden is dealt with by the patient's own immune system [113]. CD8+ T cells are the most potent immune cells capable of eliminating foreign antigens and breast tumor cells [88]. A normal microbiome contributes to maturation of effector CD8+ T cells (known as killer T cells) via contact with *Sphingomonas* species (of Proteobacteria phylum) [85]. Inflammation decreases the proportion of *Sphingomonas* in the Proteobacteria phylum and prevents proper development of CD8+ antitumor cytotoxic T cells detected in the peripheral blood [86, 87]. 

Research correlating the number of CD8+ effector T cells infiltrating breast cancer tumors with patients survival shows that patients with higher numbers of effector T cells in their breast tumors have better chance of being successfully treated for their disease or being long-term survivors than those without these immune cells. In a study of over 1,300 patients followed for over 10 years, patients whose breast tumors had more than 24 of CD8+ cells per high power field of the tumor had better breast cancer specific survival, 75% versus 45% (*P* < 0.001), than those having fewer than five [114]. In over 170 triple negative breast cancer patients followed over 8 years after the diagnosis, patients with more lymphocytes infiltrating their tumors more than 36/mm^2^ were associated with a 60% recurrence-free survival at 8 years versus 20% in patients with fewer than 20/mm^2^ (*P* < 0.0019) [115]. 

## 8. Conclusion

The incidence of breast cancer around the world is vastly different in the USA and Western Europe compared to Asia and Africa. In the industrialized world, the incidence is around 120 women per 100,000 individuals per year; in the less developed parts of the world it is 17 per 100,000 [116]. The western born children of those immigrants, however, have the same incidences of breast cancer as their western compatriots, rather than that of their ancestors. This occurs despite the obvious fact that their genes pool does not significantly change over just one generation [117]. The major change that occurred is environmental; diet and the resulting microbiome changes appear to play a major role among the possible environmental factors [118]. Advances in understanding of our diet, our microbiome, and the complex interactions between the two, hold the potential to modify not just the course of digestive diseases but also of disorders such as breast cancer. It is hoped that a deeper understanding of this world within us can point the way towards evidence based dietary therapies to decrease the risk of developing breast cancer as well as improve the outcomes of those already diagnosed. 

## Figures and Tables

**Table 1:** The composition of human colonic microbiome: phylums and selected major species belonging to each phylum [14, 17].

Phylum	Species
Actinobacteria	*Bifidobacterium *
Bacteroidetes	*Bacteroides*, *Prevotella*, *Porphyromonas *
Verrucomicrobia	*Akkermansia *
Firmicutes	*Clostridium*, *Faecalibacterium*, *Ruminococcus*, *Roseburia*, *Veillonella*, *Staphylococcus*, *Streptococcus*, segmented filamentous bacteria, *Butyrivibrio *
Tenericutes	*Erysipelotrichaceae *
Proteobacteria	*Citrobacter*, *Enterobacter*, *E. coli*, *Shigella*, *Klebsiella*, *Hemophilus*, *Sphingomonas *
Cyanobacteria	Unclassified YS2

**Table 2:** The influence of host genes on the structure of the intestinal microbiome in gnotobiotic rodents [32].

Mouse model	Phenotype/microbiome in mouse model	Systemic manifestation of abnormal microbiome in mice	Results of fecal transplant to wild type animal	References
Rag2-knockout	No functional B and T cells/inflammatory colitis	Recurrent infections	Inflammatory colitis	[23, 24]

Tbx21-knockout	No functional Th1 cells/Crohn's disease, colitis	Asthma, autoimmune disease, and various malignancies	Crohn's disease, colitis, asthma, and autoimmune disease	[25]

TLR5-knockout	No flagellin receptor	Metabolic syndrome: insulin resistance, hyperlipidemia, fat deposition on omentum, and atherosclerosis	Metabolic syndrome: insulin resistance, hyperlipidemia, fat deposition on omentum, and atherosclerosis	[26]

SHP-1 mutation	No T, B cells and no immunoglobulins/colitis	Autoimmune disease, alopecia, glomerulonephritis, pneumonitis, colitis, and paws inflammation triggered by microbiota	Colitis alopecia, glomerulonephritis, and pneumonitis	[27, 28]

NLR-P3 gene mutation	Cold urticaria inflammatory disease, dysbiosis	Dysbiosis, cold urticaria inflammatory disease	Colitis, cold urticaria inflammatory disease	[29]

NOD-2 mutation	Abnormal innate immune response	Various adenocarcinomas	Crohn's disease, dysbiosis	[30, 31]

Rag2: recombination activating gene 2.

NLR-P3: nucleotide binding oligomerization domain (NOD) like receptors P3.

Tbx21: T cell specific T-box transcription factor (crucial transcription factor for TH1 cells); TLR-5: toll-like receptor 5.

SHP-1: Src homology region 2 domain-containing phosphatase-1.

NOD-2: nucleotide binding oligomerization domain 2.

**Table 3:** Microbiome role in maturation of the immune system and involvement in cancer.

Microbiome components	Immune cell development	Role in cancer	References
Firmicutes phylum: *Fusobacterium nucleatum *	Direct killing of lymphocytes;	Promotes metastasis and tumor growth	[92]
Proteobacteria: *E. coli*, *Citrobacter*, *Enterobacter*, *Shigella*, *Klebsiella*, and *Hemophilus *	Promotes TH17	Promotes cancer progression and metastasis	[93]
Archea phylum: *Eubacterium rectale *	IL-6 production by intestinal inflammatory dendritic cells	Promotes cancer progression and metastasis	[94]
Proteobacteria phylum: *Sphingomonas* species,	CD8+ T cells	Anti cancer activities	[91, 95]
*Clostridium* cluster IV-XIVa			[22, 96–101]
Actinobacterium phylum: *Bifidobacterium *	Th1 noninflammatory	Protects against inflammation and cancer	[102]
